# Adaptation of the Mullen Scales of Early Learning for use among infants aged 5‐ to 24‐months in rural Gambia

**DOI:** 10.1111/desc.12808

**Published:** 2019-03-10

**Authors:** Bosiljka Milosavljevic, Perijne Vellekoop, Helen Maris, Drew Halliday, Saikou Drammeh, Lamin Sanyang, Momodou K. Darboe, Clare Elwell, Sophie E. Moore, Sarah Lloyd‐Fox

**Affiliations:** ^1^ Centre for Brain and Cognitive Development Birkbeck, University of London London UK; ^2^ The Global Health and Tropical Medicine Training Institute Amsterdam The Netherlands; ^3^ Department of Psychology University of Victoria Victoria BC Canada; ^4^ Medical Research Council The Gambia, at The London School of Hygiene and Tropical Medicine London UK; ^5^ Department of Medical Physics and Biomedical Engineering University College London London UK; ^6^ Department of Women and Children's Health King's College London London UK; ^7^ Department of Psychology University of Cambridge Cambridge UK

**Keywords:** cognitive development, global mental health, growth, infancy, Mullen Scales of Early Learning, The Gambia

## Abstract

Infants in low‐resource settings are at heightened risk for compromised cognitive development due to a multitude of environmental insults in their surroundings. However, the onset of adverse outcomes and trajectory of cognitive development in these settings is not well understood. The aims of the present study were to adapt the Mullen Scales of Early Learning (MSEL) for use with infants in a rural area of The Gambia, to examine cognitive development in the first 24‐months of life and to assess the association between cognitive performance and physical growth. In Phase 1 of this study, the adapted MSEL was tested on 52 infants aged 9‐ to 24‐months (some of whom were tested longitudinally at two time points). Further optimization and training were undertaken and Phase 2 of the study was conducted, where the original measures were administered to 119 newly recruited infants aged 5‐ to 24‐months. Infant length, weight and head circumference were measured concurrently in both phases. Participants from both phases were split into age categories of 5–9 m (*N = *32), 10–14 m (*N *=* *92), 15–19 m (*N *=* *53) and 20–24 m (*N *=* *43) and performance was compared across age groups. From the ages of 10–14 m, Gambian infants obtained lower MSEL scores than US norms. Performance decreased with age and was lowest in the 20–24 m old group. Differential onsets of reduced performance were observed in the individual MSEL domains, with declines in visual perception and motor performance detected as early as at 10–14 months, while reduced language scores became evident after 15–19 months of age. Performance on the MSEL was significantly associated with measures of growth.

**RESEARCH HIGHLIGHTS**

- A population‐specific adaptation of the Mullen Scales of Early Learning (MSEL) developed for use and tested in a rural area of The Gambia.
- Infants in this setting had lower MSEL scores compared to US norms and performance declined from the ages of 5‐ to 24‐months.
- Reduced performance was observed across multiple domains of cognitive development, including perceptual, motor and language scales.
- Performance on the MSEL was significantly associated with measures of physical growth.

## 1 INTRODUCTION

The United Nations Sustainable Development Goals (UN, 2015) have identified the reduction of poor child development in low‐ and middle‐income countries (LMICs) as a key priority for global health research and interventions. A particular area of concern is compromised cognitive development within these settings. Grantham‐McGregor et al. (2007) suggest that over 200 million children worldwide fail to meet their cognitive potential due to poverty, undernutrition and inadequate care. This has significant impact on individuals’ quality of life, as well as the nation's workforce and economic development (Alderman, Behrman, Grantham‐McGregor, Lopez‐Boo, & Urzua, 2014). Multiple interventions have attempted to improve cognitive outcomes of children who are at‐risk, which have had varied effectiveness (for review, see Perkins et al., 2017). Discrepancies are likely due to outstanding questions about the nature and trajectory of compromised cognitive development in LMICs. The age at which interventions are most effective and domains of cognitive functioning most at‐risk are still under investigation (Perkins et al., 2017). Moreover, the appropriateness of using established measures of cognitive development in these settings is uncertain (Isaacs & Oates, 2008).

The present study aims to address these issues by adapting a widely used measure of child development, the Mullen Scales of Early Learning (MSEL; Mullen, 1995), for use with infants in a rural area of The Gambia, West Africa. The objective is to examine the applicability and challenges of using a population‐specific adaptation of the MSEL within this context. Furthermore, we examine age‐related trajectories of cognitive development within the first 24‐months of life in this setting to identify key periods of vulnerability, across multiple domains. We also assess the association between cognitive performance and measures of growth.

### 1.1 Cognitive development in infancy and periods of sensitivity

Within the first 24‐months of life, neuronal networks develop most rapidly and exhibit high plasticity (Fox, Calkins, & Bell, 1994; Fox, Levitt, & Nelson, 2010; Kolb & Gibb, 2011). Environmental factors in early life, such as home environment, caregiver responsiveness, infection and diet, have important and lasting impacts on neural development (e.g., Murray‐Kolb et al., 2014). Consequently, the infant brain is highly vulnerable to compromised development during this time. Yet, it is also during this period that interventions may be most effective (Kolb & Gibb, 2011). Thus, many interventions targeted at reducing developmental delay are administered in the first years of life, with the aim of minimizing the impact of adverse factors before poorer developmental outcomes are fully manifest (Petrenko, 2013).

It is, however, important to note that the ontogenetic events of neural maturation occur at different periods during this early time frame and that they have cascading effects (Grantham‐McGregor et al., 2007; Thompson & Nelson, 2001). For example, visual reception (VR) begins to develop at birth and by approximately 6‐months of age, infants exhibit similar patterns of visual preference and visuospatial organization as adults (Deen et al., 2017; Emberson, Richards, & Aslin, 2015). On the other hand, advances in communicative development begin to emerge later and are most prominent by 12‐months of age (e.g., Levine, Strother‐Garcia, Golinkoff, & Hirsh‐Pasek, 2016). Yet, evidence suggests that infants’ early perceptual abilities influence the development of speech perception and production later on (Bruderer, Danielson, Kandhadai, & Werker, 2015). Similarly, healthy development of motor abilities is posited to assist infants in engaging with their environment, thus providing the opportunity to hone other cognitive skills (Iverson, 2010). Among other populations at‐risk for cognitive delay, such as infants at familial risk for autism spectrum disorder (ASD), delays in sensorimotor development emerge very early in infancy and precede atypicalities in language performance (Leonard et al., 2014; Nishimura, Takei, Tsuchiya, Asano, & Mori, 2016). Thus, we argue that it is insufficient to solely measure global cognitive development, or development during later childhood only, when trying to identify a key period of developmental risk. Rather, it is important to track age‐related changes in performance across specific perceptual, motor and language domains to observe more fine‐grained periods when development may be sensitive to insults.

### 1.2 Measuring infant development in LMICs

There is an emerging body of research that has investigated cognitive development in LMICs. This research has provided important insights into how environmental risk factors such as undernutrition, lack of stimulation, poverty and disease contribute to compromised cognitive development (Fernald, Kariger, Hidrobo, & Gertler, 2012; Fernald et al., 2006; McCoy et al., 2016). However, there is considerable variability in the types of cognitive assessment methods used, outcomes measured and age of children tested in the studies. Many studies use school performance as a measure of cognitive outcome (Fernald et al., 2012). Others use standardized measures of language and IQ (e.g., Schady et al., 2015), which provide useful information but are not suitable for young, pre‐verbal children. Thus, if studies are to measure cognitive development during early life, assessment methods suitable for this age need to be identified.

The MSEL and the Bayley Scales of Infant and Toddler Development (Bayley, 1995) are key measures used to assess child developmental level in the first 5 and 3 years of life, respectively. These assessments have been used widely in research and measure development across perceptual, motor and language domains. A key limitation of these measures is that they have been designed for and normed in Western, English‐speaking populations. Consequently, their utility in LMICs is limited without adaptation to the specific target population and language (Isaacs & Oates, 2008). Several such adaptations already exist, providing a guide for future researchers attempting to use infant development measures cross culturally.

Hamadani et al. (2014) measured the association between poverty and infant cognitive development in Bangladesh using the Bayley scales, among other measures, at 7‐, 18‐ and 64‐months of age. Their study reports that children from the lowest socioeconomic status (SES) group in the sample exhibited reduced cognitive performance from the age of 7‐months. Furthermore, the cognitive deficit observed in this group increased with age. One limitation of this study is that it did not use the same measure at all time points (Bayley scales were only used at 18‐months), leaving unclear the effectiveness and applicability using a single measure of cognitive ability for longitudinal follow‐up. Koura et al. (2013) adapted the MSEL for use with 12‐month old infants in Benin and found that a substantial proportion of children at this age exhibited developmental delays. Scores from the MSEL were also significantly associated with parent‐report measures of infant development, suggesting good validity of this adapted scale. Bodeau‐Livinec et al. (2018) utilized the MSEL that had been adapted for Benin (Koura et al., 2013) and report reasonable construct validity for both raw and standardized scores among children aged 3–6 years in this setting. Likewise, cognitive performance was significantly associated with measures of risk factors (e.g., maternal depression).

Multiple studies have adapted the MSEL and Bayley scales for use in South Africa, all reporting satisfactory utility of the scales within this setting. Ballot et al. (2017) used the Bayley scales in a longitudinal study and report that infants exhibited a decline in language scores after the age of 12‐months. In contrast, no change in global cognitive performance was detected, highlighting the importance of examining development across several age points and cognitive domains. Bornman, Sevcik, Romski, and Pae (2010) describe in detail an adaptation of the MSEL for use with Afrikaans‐speaking children in South Africa. They report that standardized scores on the MSEL corresponded with parent‐report of developmental milestones, but that no age‐related differences in these scores were observed among children aged 3–6 years. Bornman et al. (2018) assessed age‐related changes in MSEL performance in a cross‐sectional study with children aged 21‐ to 68‐months. Their study reports that MSEL scores increased with age, but it is important to note that only raw scores (and not scores corrected for age) were examined, which are expected to increase since older children complete more items. Finally, Boivin et al. (2013) report increases in MSEL performance from the ages of 6‐ to 18‐months after a parent‐led intervention, among Ugandan infants exposed to HIV. A number of these studies also report sex differences, with girls scoring higher than boys, particularly in language subscales (e.g., Koura et al., 2013).

Taken together, these studies suggest that population‐specific adaptations of key developmental assessments can be applied in LMICs. They demonstrate appropriateness for use in the contexts that they were designed for, are sensitive to changes in performance with age and are able to detect improvement after intervention. However, Perkins et al. (2017) warn that many adaptations are designed to be suitable among children of higher SES. This is evident in some of the studies described above, where the measures were translated into the country's official European language (e.g., French in Benin, Koura et al., 2013) while a large proportion of the population in these countries have a local language as their first language (Wright, 2016). Many of these adaptations have been tested in urban or semi‐urban settings, while children most highly at‐risk are likely to be living in rural areas (Baulch, 2011). Moreover, these studies did not describe in detail the challenges of tailoring and administering the adapted assessments in their respective settings. This would have been highly useful information for future research aiming to adapt infant behavioural measures for similar contexts.

### 1.3 Association between cognitive development and growth

Nutritional intake both pre‐ and postnatally is posited to have important consequences for the structural development of the infant brain, which, in turn, lays the foundations for cognitive development (Benton, 2008; Nurliyana, Mohd Shariff, Mohd Taib, Gan, & Tan, 2016). Human autopsy studies and animal models support this hypothesis, suggesting that undernutrition is associated with compromised neural composition in both infants and adults (Prado & Dewey, 2014). Furthermore, deficiencies in specific micronutrients and malnutrition, prenatally and in infancy, are associated with reduced cognitive performance and developmental delays (Nyaradi, Li, Hickling, Foster, & Oddy, 2013).

There is a growing literature that directly examines the relationship between physical growth (an indicator of nutritional status) and cognitive development in young children in LMICs. Using a meta‐analytic approach, Sudfeld et al. (2015) examined the association between pooled adjusted standardized mean performance in cognitive ability and infant length. Among children under the age of 2 years, a single unit increase in length was associated with significantly higher cognitive performance. This relationship was also observed among children older than 2 years, but to a lesser extent. This study also reports that, longitudinally, a unit increase in length under the age of 2 years is associated with improved cognitive ability at the age of 5–11 years.

The impact that nutritional interventions and “catch‐up” growth have on the reversibility of insults to cognitive development remains under investigation (Perkins et al., 2017). Numerous reports suggest that interventions involving early nutritional supplementation have shown beneficial effects on both physical growth and cognitive performance (Stewart et al., 2018; Tofail et al., 2018). Nutritional interventions have been shown to be most effective at minimizing the impact of insults to cognitive development when administered during the first 2 years of life (Ip et al., 2017). However, there is vast heterogeneity in this domain, since other studies concluded that improving nutritional intake does not result in better cognitive performance (Sokolovic et al., 2014). Perkins et al. (2017) argue that these discrepant findings could be due to heterogeneity in the type of intervention administered, child sex and family demographics. Furthermore, these associative findings are largely drawn by examining early life growth measures and later pre‐school or school aged cognitive markers. The parallel investigation of physical growth and cognitive development during early childhood would enable us to better disentangle the relative impact that early‐life risk factors have on development.

### 1.4 Context and setting of present study

The current study preceded, and contributed to, the development of a large‐scale prospective longitudinal study now underway in The Gambia to measure early brain and cognitive development across the first 2 years of life: The Brain Imaging for Global Health (BRIGHT) Study—www.globalfnirs.org/the-bright-project. This project aims to implement brain imaging and neurocognitive developmental methods (including functional near infra‐red spectroscopy [fNIRS], electroencephalography [EEG] and MSEL) to model longitudinal changes in brain function and cognitive development, identify critical time points, and moderators and mediators of compromised development within this rural Gambian population. In preparation for this large‐scale study, multiple feasibility and pilot studies have been conducted combining neuroimaging (fNIRS, EEG), behavioural (MSEL) and growth measures in a longitudinal and cross‐sectional design. Results of the early fNIRS studies have already been published (Begus et al., 2016; Lloyd‐Fox et al., 2014, 2017; Papademetriou et al., 2014). The present study describes the outcomes of a series of pilot studies using the key cognitive development behavioural assessment of the BRIGHT study—the MSEL.

This research was undertaken at the Medical Research Council (MRC) field station in the village of Keneba, in the rural West Kiang region of The Gambia (www.mrc.gm; www.ing.mrc.ac.uk). The community in this region relies predominantly on subsistence farming and, thus, eating patterns and income vary greatly between the annual wet and dry seasons (Hennig et al., 2015; van der Merwe et al., 2013). Additionally, there is a high prevalence of infectious disease (van der Merwe et al., 2013). Taken together, these factors pose important threats to healthy development, which is reflected in the finding that a majority of the children in this setting exhibit moderately severe growth faltering from the age of 3‐months (van der Merwe et al., 2013).

### 1.5 Aims and hypotheses

The aims of this study were to assess the applicability of an environmentally and linguistically adapted MSEL for use among infants aged 5‐ to 24‐months in this setting. Likewise, we sought to examine cognitive development and age‐related changes in performance on the MSEL to identify crucial periods of sensitivity in both overall cognitive performance and on individual visual, motor and language domains. Finally, we examined the association between MSEL scores and measures of growth. Specifically, we aimed to test the following hypotheses:

1. Given prior findings that early life exposure to environmental risk is associated with reduced cognitive ability from an early age, and that performance worsens with age (e.g., Hamadani et al., 2014), we predict that our sample will exhibit reduced MSEL scores (compared to US norms) from the age of 5‐months. We also expect that performance will worsen with increasing age.
2. Furthermore, since different domains of cognition develop at different periods within the first 24‐months (Thompson & Nelson, 2001), we expect that reduced performance will be apparent at different ages for individual domains. Since, perceptual and motor skills develop very early in infancy, we predict that declines in VR and motor domains will become apparent first, followed by declines in language development.
3. Finally, growth in early life has been associated with long‐term cognitive ability (e.g., Sudfeld et al., 2015) thus we predict that measures of growth will be positively associated with cognitive development in our sample. Given that reduced cognitive performance and growth faltering become more apparent with age (Perkins et al., 2017), we also predict that the strength of the association between cognitive performance and growth will increase with age.

## 2 METHOD

### 2.1 Participants

Two phases of testing were undertaken between February 2013 and April 2014 with a total of 171 children born in the West Kiang region. A total of 192 infants were recruited for the study but not all completed the assessments (see Results).

According to The Gambia Bureau of Statistics (2011), a majority of the population in this setting live below the poverty line, with an average income of below $2/day. Literacy rates, particularly among women, are low but increasing numbers of children attend primary school. However, fewer than half transition to secondary education and there is minimal opportunity to attend preschool. A typical diet consists of staple foods (e.g., white rice, millet or maize) with limited additional ingredients, such as sauce made from groundnuts, vegetable or palm oil, fish and vegetables (Dominguez‐Salas et al., 2013). Rates of exclusive breastfeeding are high, and infants are predominantly breastfed until the age of 24‐months (Eriksen et al., 2017). Weaning foods are commonly nutritionally insufficient, leading to widespread growth faltering (Nabwera, Fulford, Moore, & Prentice, 2017).

The residents of the West Kiang region have access to primary care services, provided for by the MRC, as well as local healthcare facilities run by the Gambian Ministry of Health (Hennig et al., 2015; Rayco‐Solon, Moore, Fulford, & Prentice, 2004). The MRC provides a wide range of nutrition‐specific interventions, antenatal and child care. These have led to a significant decrease in infant mortality, but undernutrition remains highly prevalent (Nabwera et al., 2017). While English is the official language in The Gambia, almost 80% of the population in the West Kiang region have Mandinka as their primary language (Hennig et al., 2015). All infants in this study came from households where Mandinka was spoken.

#### 2.1.1 Phase 1

Between February and November 2013, 66 infants (32 female, 34 male) aged 9‐ to 24‐months were recruited using the West Kiang Demographic Surveillance System (Hennig et al., 2015). All were born full‐term (37+ weeks gestation) and had normal birth weight. Within this phase, there were two groups of participants. The first group consisted of 42 infants (18 female, 24 male) who were tested longitudinally. Baseline was at age 9‐ to 17‐months and the follow‐up was conducted 3‐months later, when they were aged 12‐ to 20‐months. The second group consisted of 24 infants (14 female, 10 male), tested cross‐sectionally between the ages of 18‐ and 24‐months.

#### 2.1.2 Phase 2

Between February and April 2014, 126 infants (68 female, 58 male) aged 5‐ to 24‐months were recruited (by random selection) from the Early Nutrition and Immune Development (ENID; Moore et al., 2012) trial. These children from the ENID cohort were a subset of the full cohort and were included in this phase if they reached the age of 6‐, 12‐, 18‐ or 24‐months (±1 month) during the time of testing.

For both phases, ethics approval was granted by the joint Gambia Government/MRC Unit of The Gambia Ethics Committee. Written informed consent for participation and further use of videotapes was obtained from parents.

### 2.2 Demographic characteristics

Basic demographic data was available on all participants, collected as part of the West Kiang Demographic Surveillance System (Hennig et al., 2015). The ENID trial (Moore et al., 2012) collected more detailed socio‐demographic data from all participants aged 12‐months or older. This included maternal and paternal age at the time of the infant's birth, whether parents attended school and the duration (in years) spent in education, family annual household income and number of full siblings (with the same mother and father as target infant). For the present study, we obtained this ENID‐collected demographic data for all participants recruited from that study (in Phase 2), who were aged 12+ months at the time of testing. Other than parental age at time of birth, there were no socio‐demographic data available for participants who were younger than 12‐months in Phase 2 and socio‐demographic information was not collected for participants tested in Phase 1.

### 2.3 Measures of cognitive development

*The MSEL* (Mullen, 1995) is an assessment battery that measures child developmental level from birth to 68‐months of age. The MSEL consists of 5 subscales that measure cognitive development across the domains of VR, Fine Motor (FM) skills, Gross Motor (GM) skills, Receptive Language (RL) and Expressive Language (EL). Each subscale consists of a set of performance‐based items presented in hierarchical order of difficulty, where children are rated on whether they successfully complete the task in each item. Raw scores on each subscale can be converted to age‐normed *t*‐scores (*M *=* *50, *SD *=* *10), based on a U.S. sample. Furthermore, a global cognitive *t*‐score (*M *=* *200, *SD *=* *30; hereafter “Cog *T*”) can be computed by summing *t*‐scores from the VR, FM, RL and EL subscales. While GM scores are not included in the Cog *T* score and are not considered to constitute cognitive development, we included this scale in analyses in order to examine performance on the complete MSEL. The MSEL also provides descriptive categories that correspond to overall scores; *Very High* (Cog *T* ≥ 260), *Above Average* (Cog *T* = 231–259), *Average* (Cog *T* = 169–230), *Below Average* (Cog *T* = 138–168) and *Very Low* (Cog *T* ≤ 137). The original MSEL studies (Mullen, 1995) report that the measure has good psychometric properties across the different subscales and total score. For example, the 5 subscales exhibit satisfactory internal consistency, with median values across age groups ranging from *r *=* *0.75 to *r *=* *0.83. Similarly, the scales had high test re‐test reliability, with scores ranging from *r *=* *0.782 to *r *=* *0.85 for the cognitive scales and *r *=* *0.96 for the GM scale.

### 2.4 Linguistic translation and adaptation of the MSEL

Adaptation of the MSEL was undertaken with the help of a panel that consisted of the co‐investigator and local principal investigator (MKD) and two senior MRC field workers (SD, LS), who are all native Mandinka speakers. Specific details about the adaptations are provided in Supporting Information.

#### 2.4.1 Linguistic translation

MSEL instructions, item cues and language scales were translated into Mandinka. Peña (2007) provides guidelines for translating measures into different languages and suggests that, in addition to undertaking a pure linguistic translation, functional, cultural and metric equivalence of the translated items need to be considered. In this way, researchers can ensure that the translated measure captures subtleties in meaning, while maintaining an equivalent level of difficulty.

Given that Mandinka is not a written language and is, thus, without an official dictionary (e.g., Gamble, 1987), there can be substantial variability in both spoken and written Mandinka. Therefore, translation was undertaken in collaboration between the three panel members and a consensus translation was reached for each item. Subsequently, an additional, Mandinka‐speaking, field assistant, reviewed the translated MSEL to assess accuracy and word choice. Where discrepancies were noted, the field assistant provided the panel with suggestions for alternatives. The panel reviewed these suggestions and made necessary adjustments to develop an updated translation. Finally, the updated version underwent blind‐back translation to English by a field assistant who was not involved in the original translation. The back translated version was compared to the original English MSEL by the panel to assess accuracy. Discrepancies were discussed among the panel members and resolved by committee consensus.

As outlined by Peña (2007), the linguistic and cultural differences between English and Mandinka did not allow for pure linguistic equivalence in translation. Instead, the aim was to achieve functional and cultural equivalence. The phrasing of item cues was altered to adhere to local speaking patterns. When changing the phrasing, care was taken to ensure that the intended outcome of the instructions remained the same. For example, some items ask the participant to identify a toy baby doll by telling them “pat baby,” in Mandinka we asked the children to “take baby” because requesting a child to pat a doll is less common in that setting. Due to the stark environmental differences, it was not always possible to achieve cultural equivalence and certain stimuli had to be replaced (see below). Finally, efforts were made to maintain metric equivalence of the two measures by ensuring that the adapted material had words and instructions that had the similar difficulty, length, frequency of use and level of guidance as in the English‐language version.

#### 2.4.2 Substitution of culturally inappropriate stimuli and materials

Toys and stimuli that were deemed inappropriate or unfamiliar to children in this setting were identified. These included items that were not commonly observed (e.g., a TV) or were visually different (e.g., a Western style house) in the rural Gambian context, or were objects that children were reluctant to engage with (e.g., a light‐skinned doll). When selecting replacements for these stimuli, efforts were made to maintain the category of the item (e.g., household object, animal). The Supporting Information describes the adaptations in full detail.

#### 2.4.3 Training

Field workers, already familiar with gross and fine motor assessments of infants and toddlers, were trained in MSEL administration by experienced researchers from the UK (SLF, PV, HM). Training consisted of watching videos and live demonstrations of item administration and discussion of scoring criteria. Trainees were given opportunity to rehearse administration with each other first. Subsequently, they administered the assessment to child participants. Supervision was provided both by their peers in the training group and supervisors. Testing sessions were video‐recorded, and trainees were given in‐depth feedback on their administration and scoring. A second phase of refresher training was conducted prior to data collection for Phase 2.

### 2.5 Anthropometric measures

Measurement of length, weight and head circumference (HC) was performed on all infants. Measurements were taken by trained field workers using calibrated tools. Length was measured using a Harpenden Infantometer length board (Holtain Ltd) to a precision of 0.1 cm. Weight was obtained using an electronic baby scale (Model 336, SECA) to a precision of 0.01 kg. Finally, HC was measured around the maximum circumference of the head (forehead to occiput) using stretch‐proof measuring tape (Model 201, SECA) to the nearest 0.1 cm. Each measure was taken in triplicate and the mean of the three measures was used in analyses.

Anthropometric measures were converted to age and sex adjusted *z*‐scores that are based on World Health Organization normative growth data (WHO Multicentre Growth Reference Study Group, 2006). Height‐for‐Age (HAZ), Weight‐for‐Height (WHZ) and Head Circumference (HCZ) *z*‐scores were computed. Children categorized as “stunted” or “wasted” were identified using WHO criteria based on HAZ and WHZ scores, respectively. Severity of stunting/wasting is categorized as −2 *SD* for moderate and −3 *SD* for severe.

### 2.6 Procedure and optimization

#### 2.6.1 Testing procedure

Children were tested in the morning in a quiet room. Breakfast was provided for all participants to minimize risk of hunger or fatigue. In the case of infant refusal or disinterest in the task, testing was interrupted and continued later in the morning. Caregivers were present during all testing sessions and were asked to positively encourage their children during testing if they were hesitant to interact with the administrator.

Each participant was tested by a single field assistant, who underwent the aforementioned training, and all sessions were video‐recorded. Additionally, one of the researchers from the UK (SLF, HM, PV), who are all trained on the MSEL and have 3–5 years of experience in administration, was present during every testing session. Scoring of the assessments was done concurrently by both the administrator and the researcher present. Consensus on the scores was compared after administration and, in the case of incongruent scoring between assessors, tapes were reviewed, and mutual agreement was reached. Assessors were also instructed to record all items where the infant refused to respond and all items that were scored based on parent‐report rather than task administration. A similar scoring method, whereby the assessment is concurrently scored by an examiner and a trained observer, has been applied in a variety of research using behavioural measures with infants (e.g., Gammer et al., 2015).

#### 2.6.2 Optimization

After Phase 1 of data collection, it became apparent that a substantial number of items were completed using parent‐report for items where this was not a valid response option. Thus, the administrators underwent additional training, to reduce parental report before commencing Phase 2 of testing. Additionally, the children's reactions to the adapted stimuli in Phase 1 were reviewed and any stimuli or toys that were found to be inappropriate during testing were re‐evaluated and replaced by more suitable ones for use in Phase 2.

### 2.7 Analyses

Participants from Phases 1 and 2 were analysed as one combined group and divided into four age categories: 5–9 months, 10–14 months, 15–19 months and 20–24 months. Data collected from participants who were tested longitudinally was included separately in the age category that corresponded with their age at the baseline and follow‐up visits.

The proportion of males and females in each age category was evaluated using chi‐square analyses. Furthermore, given prior reports of sex differences in cognitive assessments (e.g., Koura et al., 2013), we tested for sex differences in our sample using analysis of variance (ANOVA) to compare Cog *T* and all subscale *t*‐scores across sex. Significant sex differences emerged (see Results), thus sex was controlled for in all further analyses.

Socio‐demographic characteristics obtained from the ENID trial (Moore et al., 2012) in Phase 2 were compared between the age groups using ANOVA and chi‐square, where appropriate.

Likewise, differences in Cog *T* and subscale *t*‐scores in the two phases of testing were compared, using ANOVA. Again, significant differences emerged between the two phases (see Results) and phase was also controlled for in further analyses.

The number of items completed by parent‐report was compared between the two phases. Given that participants do not always complete the same number of items on the MSEL, a ratio of items scored through parent‐report out of the total number of items completed was computed. These scores then underwent arcsine transformation and were compared between Phases 1 and 2 using the Mann–Whitney *U* test. To determine whether parent‐report had an impact on the total score, Spearman Rho correlation was run between number of items scored by parent‐report and Cog *T* scores.

#### 2.7.1 Comparison of Gambian MSEL scores and US norms

To assess the first hypothesis, that the Gambian sample would have reduced MSEL scores, one‐sample *t* tests were used to compare Cog *T* scores from our sample with US‐normed Cog *T* scores (*M *=* *200, *SD *=* *30). These were performed separately for each age group and Bonferroni adjusted *p*‐value (0.05/4 = 0.001) was used to account for multiple testing.

Likewise, we compared the number of participants who were in the different descriptive categories in each age group. To reduce the number of tests, the descriptive categories were collapsed to create categories that comprise normative scores (Very High, Above Average and Average; hereafter *Average and Above*) and those that indicated delay (Below Average and Very Low, hereafter *Below Average*). The proportion of participants that fell into these categories in each age group was assessed using chi‐square analysis. Post hoc tests were performed following the method suggested by Beasley and Schumacker (1995), where the adjusted standardized residuals were analysed and converted to *p*‐values. Bonferroni adjusted *p*‐values (0.05/4 = 0.001) were used to account for multiple testing.

#### 2.7.2 Age‐related changes in MSEL performance

To assess for age‐related changes in MSEL scores, Multivariate ANOVA (MANOVA) was performed to compare Cog *T* and subscale *t*‐scores across the four age groups, controlling for phase of testing and sex. Where significant differences emerged, pairwise comparisons were used to identify age groups that significantly differed from each other in each of the scales. Bonferroni correction was applied to correct for multiple testing.

To further examine age‐related trajectories in MSEL scores, additional analyses were performed in the group that was tested longitudinally. Repeated‐measures ANOVA was used to compare Cog *T* and all subscale *t*‐scores between the baseline and follow‐up visits, controlling for infant sex.

We also examined change in descriptive category between the two visits in the longitudinal group. McNemar's test was used to compare the proportion of participants that had shifted between the *Average and Above* and *Below Average* categories from baseline to follow‐up. When a significant result emerged, post hoc analyses were done by performing McNemar's test in the *Average and Above* and *Below Average* categories separately, with Bonferroni adjusted *p*‐values (0.05/2 = 0.01) to account for multiple testing.

#### 2.7.3 Association between MSEL scores and measures of growth

Age and sex differences in the growth measures were assessed first. MANOVA was used to compare mean WHZ, HAZ and HCZ across the 4 age groups and between males and females. Where significant differences emerged, pairwise comparisons were performed to identify which groups significantly differed from each other. Bonferroni correction was applied to account for multiple testing.

Subsequently, we sought to examine the association between Cog *T* scores WHZ, HAZ and HCZ. Spearman Rho correlations were performed between Cog *T* scores and each of the growth *z*‐scores to identify significant associations. Significant correlations emerged between Cog *T* and WHZ and HAZ scores (see Results). These correlations were followed up by linear regression to determine whether the associations would remain when taking into account participant age and if the strength of these associations increased as children became older. Two separate regressions were run with WHZ and HAZ as predictors in each. Cog *T* scores were entered as the dependent variable, WHZ/HAZ, age and an interaction term between age × WHZ/age × HAZ were entered as predictors. WHZ, HAZ and age were mean centred to reduce the risk of multicollinearity. Furthermore, sex and phase of testing were controlled for in both regressions.

All analyses were performed in SPSS Version 24 (IBM Corp, 2016). Effect sizes are reported as Cohen's *d*, η^2^, and *r* ^*2*^, where appropriate.

## 3 RESULTS

### 3.1 Demographic characteristics

Table 1 summarizes the ages and sex ratio of the entire sample by age group, as well as within phases. From the overall sample, 21 participants did not complete the MSEL due to refusal to participate on the day of testing. Phase 1 had data missing from 13 participants, 8 of these were among participants tested longitudinally (5 missed the baseline visit, 2 missed the follow‐up and 1 did not complete either visit) and 5 participants who were tested cross‐sectionally refused to participate. In Phase 2, only eight participants did not complete the MSEL. There were no significant differences in the proportion of males and females in Phases 1 and 2 (χ^2^ **=* *1.36, *p *=* *0.24) or across the 4 age groups (χ^2^(3)* *=* *5.61, *p *=* *0.13).

**Table 1:** Age, sex and size of the Gambian sample in each age group and Phases 1 and 2

Age/phase	*N*	Mean age in months (*SD*)	Number of boys:girls	Percentage (M, F)
5–9 m	32	6.36 (5.16)	22:10	68.75% M, 31.25% F
10–14 m	92	12.44 (1.17)	47:45	51.08% M, 48.92% F
15–19 m	53	17.62 (1.11)	23:30	43.39% M, 56.61% F
20–24 m	43	23.74 (0.94)	20:23	46.51% M, 53.49% F
Phase 1	52	14.29 (3.50)	29:23	55.77% M, 44.23% F
Phase 2	119	15.55 (6.77)	54:65	45.38% M, 54.62% F

In Phase 2 (see Table 2), there were significant differences in maternal age between the four age groups, with mothers of infants in the 20–24 m group being older when the infant was born than mothers of infants in both the 5–9 m (*p *<* *0.001, *d *=* *1.09) and 10–14 m (*p *=* *0.02, *d *=* *0.83) groups. Likewise, fathers of infants in the 20–24 m group were older when the infant was born than were fathers in the 5–9 m group (*p *=* *0.02, *d *=* *1.01). Additionally, infants in the 20–24 m group had more siblings than those in the 10–14 m group (*p *=* *0.04, *d *=* *0.98). There were also trend‐level differences between the age groups in the number of fathers who attended school (χ^2^(2)* *=* *5.24, *p *=* *0.07). There were no significant differences in any of the other socio‐demographic variables.

**Table 2:** Socio‐demographic characteristics of participants in Phase 2

Family characteristics	5–9 m *M* (*SD*)	10–14 m *M* (*SD*)	15–19 m *M* (*SD*)	20–24 m *M* (*SD*)	ANOVA/χ^2^
Maternal age (years) at birth of target child	*N *=* *27 27.78 (8.23)	*N *=* *31 30.28 (6.74)	*N *=* *27 30.92 (7.29)	*N *=* *32 35.61 (6.02)	*F*(3, 113) = 6.53, *p *<* *0.001, η^2^ = 0.15
Paternal age (years) at birth of target child	*N *=* *18 41.71 (10.75)	*N *=* *25 45.38 (11.54)	*N *=* *21 48.36 (9.32)	*N *=* *31 51.77 (9.20)	*F*(3, 91) = 3.54, *p *=* *0.02, η^2^ = 0.11
Number of mothers attended school	N/A	*N *=* *12 4	*N *=* *20 8	*N *=* *30 11	χ^2^(2) = 0.15, *p *=* *0.93
Duration of maternal education (years)	N/A	*N *=* *4 4.25 (1.71)	*N *=* *8 3.88 (2.42)	*N *=* *11 3.91 (2.43)	*F*(2, 20) = 0.04, *p *=* *0.96, η^2^ = 0.004
Number of fathers attended school	N/A	*N *=* *12 4	*N *=* *18 8	*N *=* *28 4	χ^2^(2) = 5.24, *p *=* *0.07
Duration of paternal education (years)	N/A	*N *=* *4 7.50 (1.91)	*N *=* *8 6.25 (3.58)	*N *=* *4 5.25 (2.63)	*F*(2, 13) = 0.55, *p *=* *0.59, η^2^ = 0.08
Number of full siblings	N/A	*N *=* *12 4.25 (2.09)	*N *=* *20 5.05 (2.49)	*N *=* *30 6.50 (2.49)	*F*(2, 59) = 3.90, *p *=* *0.03, η^2^ = 0.12
Total family income (Dalasi/year)	N/A	*N *=* *12 20,208.33 (12,948.25)	*N *=* *19 15,763.16 (14,570.11)	*N *=* *28 13,071.43 (7,816.80)	*F*(2, 56) = 1.18, *p *= 0.31, η^2^ = 0.04

*Note*

Data on parental education, number of siblings and family income is only available for infants aged over 12 months within the 10‐ to 14‐month group. Furthermore, this data is not available for participants in the 5‐ to 9‐month age group.

Significant sex differences were observed in the Cog *T* score (*F*(1, 207)* *=* *7.41, *p *=* *0.01, *d *=* *0.36) and the GM (*F*(1, 207)* *=* *8.73, *p *=* *0.003, *d *=* *0.41), VR (*F*(1, 207)* *=* *5.42, *p *=* *0.01, *d *=* *0.32) and FM (*F*(1, 207)* *=* *8.18, *p *=* *0.01, *d *=* *0.40) scales. In each of these subscales, males outperformed females (see Table 3 for summary of scores). Furthermore, males scored higher than females in the RL scale, but this only reached trend‐level significance (*F*(1, 207)* *=* *3.15, *p *=* *0.08, *d *=* *0.24). Because of these significant sex differences, sex was controlled for in further analyses of MSEL scores.

**Table 3:** Summary of Mullen Scales of Early Learning mean scores and standard deviations across sex and phase of testing

MSEL subscale	Male (*N *=* *106)	Female (*N *=* *103)	MANOVA group effects	Phase 1 (*N *=* *92)	Phase 2 (*N *=* *117)	MANOVA group effects
Mean Cog *T* (*SD*)	179.92 (30.27)	168.47 (30.52)	*F*(1, 207) = 7.41, *p *=* *0.01, *d *=* *0.36	180.26 (33.16)	169.56 (30.86)	*F*(1, 207) = 6.35, *p *=* *0.01, *d *=* *0.35
Mean GM (*SD*)	50.17 (12.36)	44.94 (13.22)	*F*(1, 207) = 8.73, *p *=* *0.003, *d *=* *0.41	51.55 (14.17)	44.48 (11.16)	*F*(1, 207) = 16.32, *p *<* *0.001, *d *=* *0.55
Mean VR (*SD*)	45.45 (11.10)	41.83 (11.43)	*F*(1, 207) = 5.42, *p *=* *0.01, *d *=* *0.32	43.52 (11.74)	43.78 (11.15)	*F*(1, 207) = 0.03, *p *=* *0.87, *d *=* *0.002
Mean FM (*SD*)	49.59 (11.33)	45.39 (9.85)	*F*(1, 207) = 8.18, *p *=* *0.01, *d *=* *0.40	50.87 (12.01)	44.89 (8.97)	*F*(1, 207) = 16.98, *p *<* *0.001, *d *=* *0.56
Mean RL (*SD*)	42.39 (10.37)	39.89 (9.92)	*F*(1, 207) = 3.15, *p *=* *0.08, *d *=* *0.25	42.30 (9.92)	40.26 (10.38)	*F*(1, 207) = 2.09, *p *=* *0.15, *d *=* *.0.20
Mean EL (*SD*)	42.48 (9.16)	41.36 (10.70)	*F*(1, 207) = 0.67, *p *=* *0.42, *d *=* *0.11	43.57 (9.65)	40.64 (10.02)	*F*(1, 207) = 4.53, *p *=* *0.03, *d *=* *0.30

*Note*

MSEL: Mullen Scales of Early Learning; Cog *T*: cognitive *t*‐score; GM: Gross Motor; VR: Visual Reception; FM: Fine Motor; RL: Receptive Language; EL: Expressive Language.

Significant differences were also observed between the two phases of testing in the Cog *T* score (*F*(1, 207)* *=* *6.35, *p *=* *0.01, *d *=* *0.35), the GM scale (*F*(1, 207) = 16.32, *p *<* *0.001, *d *=* *0.55), the FM scale (*F*(1, 207)* *=* *16.98, *p *<* *0.001, *d *=* *0.56) and the EL scale (*F*(1, 207)* *=* *4.53, *p *=* *0.03, *d *=* *0.30). Scores were higher in Phase 1 for every scale where a significant difference was detected (Table 3). Consequently, phase was controlled for in all further analyses.

In Phase 1, an average of 17.2% (*SD *=* *16.1) of items were completed using parent‐report, while in Phase 2 this decreased to 3.6% (*SD *=* *2.98). This decrease was statistically significant, *U *=* *1282.50, *p *<* *0.001. On the other hand, the number of items that were completed using parent‐report was not significantly associated with Cog *T* scores, *r*_s_(209)* *=* *−0.06, *p *=* *0.43.

### 3.2 Comparison of Gambian MSEL scores and US norms

**Figure 1:**
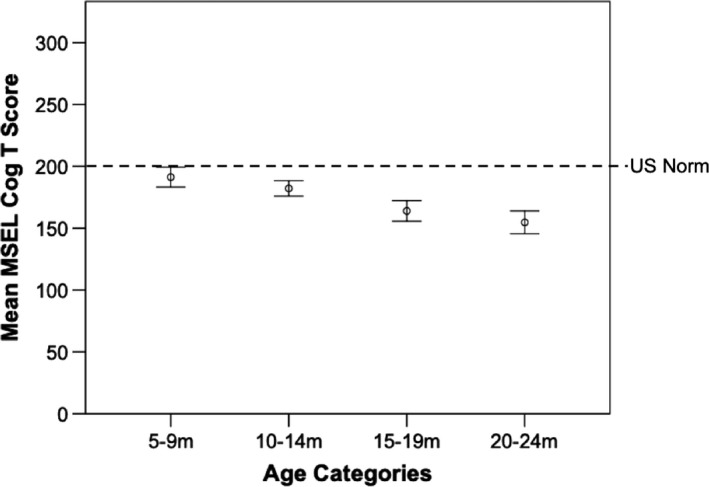
Mean Mullen Scales of Early Learning (MSEL) Cognitive *t*‐scores in each age group (5–9 m, 10–14 m, 15–19 m, 20–24 m) compared to the US norm. Error bars represent 95% confidence intervals

**Figure 2:**
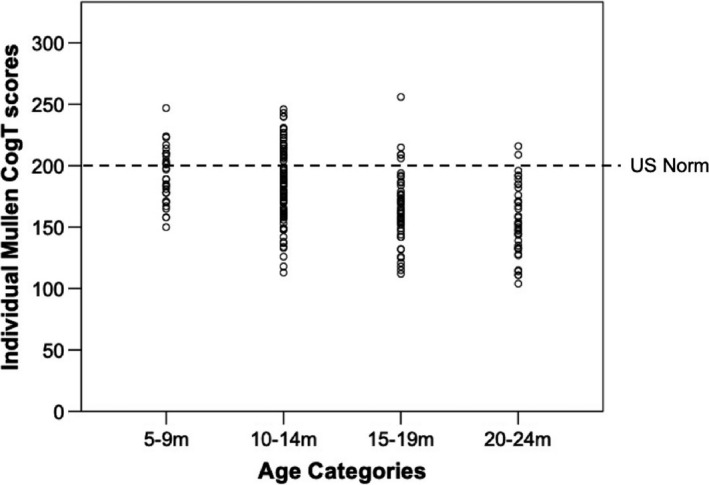
Individual data points for Mullen Scales of Early Learning Cognitive *t*‐scores in each age group (5–9 m, 10–14 m, 15–19 m, 20–24 m) compared to the US norm

At 5–9 m there was no significant difference between the Cog *T* scores obtained by the Gambian sample and the US norms (*t*(31)* *=* *−2.20, *p *=* *0.04, *d *=* *0.33), with a Bonferonni‐corrected *p*‐value applied. However, at the ages of 10–14 m (*t*(89)* *=* *−5.69, *p *<* *0.001, *d *=* *0.60), 15–19 m (*t*(47)* *=* *−8.74, *p *<* *0.001, *d *=* *1.23) and 20–24 m (*t*(38)* *=* *−9.96, *p *<* *0.001, *d *=* *1.55) the scores obtained from the Gambian infants were significantly lower than the US norms (see Figure 1 for summary of mean scores at each age point and Figure 2 for individual participant scores).

Furthermore, examining the proportion of participants that were *Average and Above* and *Below Average* revealed significant differences between the age groups. At 5‐ to 9‐months, there was a significantly greater proportion of participants scoring *Average and Above* (84.38%) than *Below Average* (15.63%), *p *<* *0.001. At 10‐ to 14‐months, a larger number of participants scored *Average or Above* (66.67%) than *Below Average* (33.33), however this did not reach significance after Bonferroni correction (*p *=* *0.02). By 15‐ to 19‐months, a significantly greater portion of participants (58.33%) scored *Below Average* than those scoring *Average or Above* (41.67%), *p *=* *0.01. At 20‐ to 24‐months, this pattern was similar, with a significantly greater percentage of participants scoring *Below Average* (66.67%) compared with those who scored *Average or Above* (33.33%), *p *<* *0.001.

### 3.3 Age‐related changes in MSEL performance

**Figure 3:**
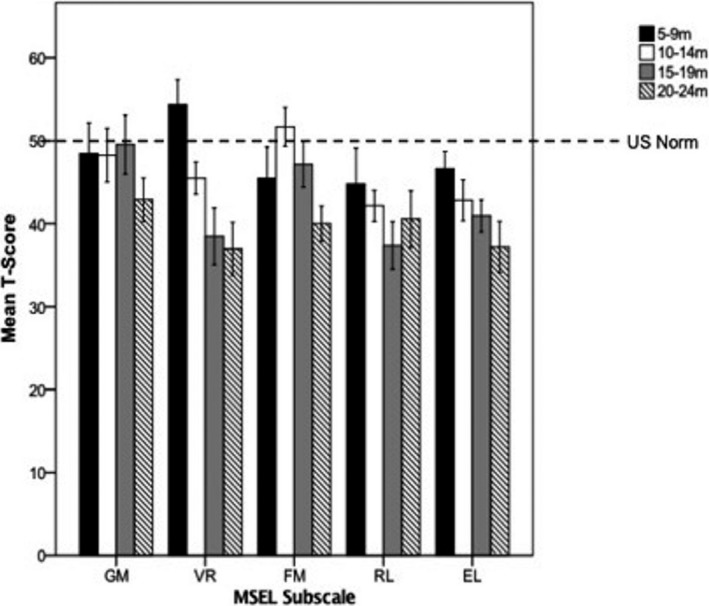
Mullen Scales of Early Learning (MSEL) subscale *t*‐scores for each age category with reference to the US norm scores (*M* = 50, *SD* = 10). Error bars represent 95% confidence intervals

Table 4 shows a summary of the MSEL scores in each age group. After controlling for sex and phase of testing, there were significant differences in the Cog *T* (*F*(1, 203)* *=* *12.40, *p *<* *0.001, η^2^ = 0.16), EL (*F*(1, 203)* *=* *5.95, *p *=* *0.001, η^2^ = 0.08), RL (*F*(1, 203)* *=* *3.86, *p *=* *0.01, η^2^ = 0.05), FM (*F*(1, 203)* *=* *8.92, *p *<* *0.001, η^2^ = 0.12) and VR (*F*(1, 203)* *=* *22.26, *p *<* *0.001, η^2^ = 0.25) scales. Pairwise comparisons revealed multiple group differences within each of the scales. Figure 3 shows the MSEL subscale scores at each age, with reference to the US‐normed scores.

**Table 4:** Summary of the means and standard deviations of the raw scores and *t*‐scores on the Mullen Scales of Early Learning total and subscales for each age group

Subscale (raw/*t*‐score)	5–9 m (*N *=* *32)	10–14 m (*N *=* *90)	15–19 m (*N *=* *48)	20–24 m (*N *=* *39)	MANOVA group effects
Mean total raw (*SD*)	40.06 (9.39)	69.08 (9.48)	89.17 (10.18)	105.41 (11.08)	
Mean Cog *T* (*SD*)	191.31 (22.39)^a^	182.17 (29.74)^a^	163.98 (28.57)^b^	154.74 (28.37)^b^	*F*(3, 203) = 12.40, *p *<* *0.001, η^2^ = 0.16
Mean GM raw (*SD*)	9.16 (2.36)	16.01 (3.61)	21.23 (3.14)	23.72 (2.20)	
Mean GM *t*‐score (*SD*)	48.47 (10.27)	48.27 (15.46)	49.56 (12.33)	42.90 (8.05)	*F*(3, 203) = 1.54, *p *=* *0.21, η^2^ = 0.02
Mean VR raw (*SD*)	9.50 (2.26)	14.54 (2.08)	17.90 (3.48)	21.69 (3.39)	
Mean VR *t*‐score (*SD*)	54.41 (8.27)^a^	45.50 (9.27)^b^	38.50 (11.76)^c^	36.97 (9.82)^c^	*F*(3, 203) = 22.26, *p *<* *0.001, η^2^ = 0.25
Mean FM raw (*SD*)	7.81 (2.52)	15.29 (2.40)	19.38 (2.22)	21.87 (1.64)	
Mean FM *t*‐score (*SD*)	45.47 (10.55)	51.70 (11.16)^a^	47.17 (9.50)^a^	40.00 (6.51)^b^	*F*(3, 203) = 8.92, *p *<* *0.001, η^2^ = 0.12
Mean RL raw (*SD*)	7.03 (2.49)	12.26 (1.87)	15.83 (2.92)	20.69 (3.77)	
Mean RL *t*‐score (*SD*)	44.78 (12.08)^a^	42.14 (9.01)	37.38 (9.84)^b^	40.56 (10.47)	*F*(3, 203) = 3.86, *p *=* *0.01, η^2^ = 0.05
Mean EL raw (*SD*)	6.56 (1.41)	10.98 (2.70)	14.83 (2.00)	17.44 (3.65)	
Mean EL *t*‐score (*SD*)	46.66 (5.75)^a^	42.82 (11.79)	40.94 (6.60)^b^	37.21 (9.45)^b^	*F*(3, 203) = 5.95, *p *=* *0.001, η^2^ = 0.08

*Note*

Groups marked with different subscript letters (a, b, c) differed significantly with Bonferroni correction applied (*p *<* *0.005).

Cog *T*: cognitive *t*‐score; GM: Gross Motor; VR: Visual Reception; FM: Fine Motor; RL: Receptive Language; EL: Expressive Language.

On the Cog *T* scores, the 5–9 m group had significantly higher scores than both the 15–19 m (*p < *0.001, *d *=* *1.06) and 20–24 m (*p *<* *0.001, *d *=* *1.43) groups. Similarly, the 10–14 m group also had better performance than both the 15–19 m (*p *=* *0.01, *d *=* *0.62) and 20–24 m (*p *<* *0.001, *d *=* *0.94) groups.

On the VR scale, the 5–9 m group scored significantly higher than the 10–14 m (*p *=* *0.002, *d *=* *1.01), 15–19 m (*p *<* *0.001, *d *=* *1.57) and 20–24 m (*p *<* *0.001, *d *=* *1.92) groups. Likewise, the 10–14 m group scored higher than the 15–19 m (*p *=* *0.001, *d *=* *0.65) and 20–24 m (*p *<* *0.001, *d *=* *0.88) groups. On the FM scale, both the 10–14 m (*p *<* *0.001, *d *=* *1.28) and 15–19 m (*p *=* *0.02, *d *=* *0.88) groups had higher scores than the 20–24 m olds.

Finally, on the RL subscale, the 5–9 m old group scored significantly higher than the 15–19 m olds (*p *=* *0.01, *d *=* *0.67), but no other significant effects emerged. On the EL scale, the 5–9 m olds scored significantly higher than both the 15–19 m (*p *=* *0.02, *d *=* *0.92) and the 20–24 m (*p *<* *0.001, *d *=* *1.21) groups.

### 3.4 Longitudinal changes in MSEL performance

Repeated‐measures ANOVA (controlling for infant sex) in the sample that was tested longitudinally revealed that there were significant declines in MSEL performance with age, in both the Cog *T* and individual subscale scores. Table 5 summarizes the MSEL scores at the baseline and follow‐up visits. The baseline MSEL scores were significantly higher than the follow‐up for the Cog *T* (*F*(1, 32)* *=* *5.50, *p *=* *0.03, *d *=* *0.65), VR (*F*(1, 32)* *=* *4.51, *p *=* *0.04, *d *=* *0.57) and RL (*F*(1, 32)* *=* *16.30, *p *<* *0.001, *d *=* *1.18) scales.

**Table 5:** Summary of mean age (in months), sex ratio and MSEL mean *t*‐scores (*SD*) at baseline and follow‐up for the children which were tested longitudinally

	Baseline	Follow‐up	Repeated measures ANOVA
Mean age (*SD*)	11.45 (1.25)	14.23 (1.21)	
Sex (M:F)	22:15	22:17	
Mean Cog *T* (*SD*)	195.68 (28.19)	175.06 (34.49)	*F*(1, 32) = 5.50, *p*.* *=* *03, *d *=* *0.65
Mean GM (*SD*)	51.53 (12.55)	53.56 (17.05)	*F*(1, 32) =2.37, *p *=* *0.13
Mean VR (*SD*)	47.88 (9.94)	41.50 (12.46)	*F*(1, 32) = 4.51, *p*.* *=* *04, *d *=* *0.57
Mean FM (*SD*)	53.82 (13.52)	52.06 (9.97)	*F*(1, 32) = 0.44, *p *=* *0.51
Mean RL (*SD*)	48.18 (7.41)	38.06 (9.57)	*F*(1, 32) = 16.30, *p*.* *<* *0.001, *d *=* *1.18
Mean EL (*SD*)	45.79 (11.47)	43.44 (8.59)	*F*(1, 32) = 0.17, *p *=* *0.68

*Note*

Cog *T*: cognitive *t*‐score; GM: Gross Motor; VR: Visual Reception; FM: Fine Motor; RL: Receptive Language; EL: Expressive Language.

Subsequently, the number of participants in the categories of *Average and Above* and *Below Average* was examined. At Visit 1, 29 participants (85.29%) scored *Average or Above*, while 5 participants (14.71%) scored *Below Average*. At Visit 2, 19 participants (55.88%) scored *Average or Above*, while 15 scored (44.12%) scored *Below Average*.

McNemar's test was used to compare the number of participants that had changed category between *Average and Above* and *Below Average* from Visit 1 to Visit 2, the overall test was significant, *p = *0.01. Post hoc analyses revealed that 11 participants who were *Average of Above* at Visit 1 were categorized as *Below Average* at Visit 2, *p *=* *0.01. On the other hand, only one participant that was *Below Average* at Visit 1 moved up to *Average and Above* at Visit 2 and this did not reach statistical significance (*p *=* *1.00).

### 3.5 Association between MSEL scores and measures of growth

Table 6 presents a summary of the anthropometric measures in each of the age groups. The children showed significant faltering across the period of study, with significantly higher WHZ (*F*(3, 216)* *=* *4.98, *p *=* *0.02, η^2^ = 0.07), HAZ (*F*(3, 216)* *=* *2.86, *p *=* *0.04, η^2^ = 0.04) and HCZ (*F*(3, 216)* *=* *3.48, *p *=* *0.02, η^2^ = 0.05) at the earlier time points. Pairwise comparisons revealed that in the WHZ measures, the 15–19 m group had significantly lower *z*‐scores than the 5–9 m (*p *=* *0.002, *d *=* *0.83) and 10–14 m (*p *=* *0.03, *d *=* *0.32) groups. For HAZ measures, the 5–9 m group had significantly higher *z*‐scores than the 10–14 m group (*p *=* *0.04, *d *=* *0.53). Finally, in the HCZ measures, the 5–9 m group had significantly higher *z*‐scores than the 10–14 m group (*p *=* *0.01, *d *=* *0.63).

**Table 6:** Summary of anthropometric measures in the different age groups

	5–9 m	10–14 m	15–19 m	20–24 m
*M* (*SD*) weight (kg)	7.3 (0.82)	8.4 (1.34)	9.0 (1.19)	10.2 (1.17)
Mean WHZ (*SD*)	−0.12 (1.3)	−0.55 (1.4)	−1.16 (1.2)	−0.74 (0.94)
% Wasted	6.3	14.1	20.8	9.3
*M* (*SD*) Length (cm)	65.7 (2.7)	71.9 (3.4)	77.9 (3.3)	82.3 (3.1)
Mean HAZ (*SD*)	−0.69 (1.3)	−1.40 (1.4)	−1.14 (1.1)	−1.36 (1.0)
% Stunted	12.5	32.6	20.8	25.6
*M* (*SD*) HC (cm)	42.3 (1.0)	44.0 (1.3)	45.5 (1.5)	46.3 (1.1)
Mean HCZ (*SD*)	−0.58 (0.87)	−1.2 (1.1)	−0.89 (0.99)	−0.97 (0.85)

*Note*

WHZ: weight‐for‐height *z*‐score; HAZ: height‐for‐age *z*‐score; HC: head circumference; HCZ: head circumference *z*‐score.

There were significant sex differences in HCZ scores (*F*(1, 224)* *=* *7.65, *p *=* *0.01, η^2^ = 0.03), with girls having higher scores across age (*M *=* *−0.81, *SD *=* *0.95) than boys (*M *=* *−1.16, *SD *=* *0.99), *d *=* *0.36. There was also a trend‐level sex difference in HAZ (*F*(1, 224)* *=* *2.89, *p *=* *0.09, η^2^ = 0.01), where girls (*M *=* *−1.06, *SD *=* *1.13) had higher scores than boys (*M *=* *−1.34, *SD *=* *1.37), *d *=* *0.22. On the contrary, there was no significant difference in WHZ (*F*(1, 224)* *=* *1.27, *p *=* *0.26, η^2^ = 0.01).

Correlation analysis revealed a significant association between Cog *T* scores and HAZ (*r*_s_(209)* *=* *0.21, *p *=* *0.002) and WHZ (*r*_s_(209)* *=* *0.17, *p *=* *0.02). On the other hand, there was no association between HCZ and Cog *T* scores *r*_s_(209)* *=* *−0.01, *p *=* *0.92.

Regression analysis revealed that, after adjusting for sex and phase of testing, both age (β* *=* *−0.36, *t*(203)* *=* *−5.81, *p *<* *0.001) and HAZ (β* *=* *0.26, *t*(203)* *=* *4.05, *p *<* *0.001) significantly predicted Cog *T* performance. The HAZ and age interaction showed trend‐level significance (β* *=* *0.11, *t*(203)* *=* *1.70, *p *=* *0.09). Similarly, in the model where WHZ was used as a predictor, both age (β* *=* *−0.35, *t*(203)* *=* *−5.39, *p *<* *0.001) and WHZ (β* *=* *0.16, *t*(203)* *=* *2.35, *p *=* *0.02) significantly predicted Cog *T* scores, while the interaction between age and WHZ did not (β* *=* *0.02, *t*(203)* *=* *0.34, *p *=* *0.74).

## 4 DISCUSSION

The present study was the first to implement a population‐specific, tailored version of the MSEL for use with infants in a rural area of The Gambia. The sample, aged 5‐ to 24‐months, obtained lower MSEL scores than US norms and scores worsened with increasing age. Additionally, a significant association emerged between measures of growth, specifically height‐for‐age and weight‐for‐height, and cognitive performance. These findings suggest that the adapted MSEL was sensitive enough to detect age and risk‐related trajectories of development within this sample. Furthermore, with these findings, we contribute to a growing body of research, which suggests that infants growing up in LMICs are at heightened risk for adverse cognitive outcomes.

### 4.1 Performance of the Gambian sample and change in scores across age

The difference between the Gambian infants’ MSEL scores and US norms was observed as early as 10‐ to 14‐months of age and continued to be evident through to 24‐months. These findings are consistent with reports in prior literature (Fernald et al., 2012; Hamadani et al., 2014; Koura et al., 2013), which suggest that infants growing up in low‐resource environments begin to exhibit reduced cognitive ability within the first year of life. Similarly, we observed that a substantial proportion of participants in the older age categories (15‐ to 24‐months) had MSEL scores that would be categorized as below average, suggesting that these children may be at‐risk for developmental delay.

When MSEL performance was compared across age groups, there was a significant decline in scores on both global cognitive performance and on individual subscales. Importantly, the decline in overall cognitive ability started to be evident in the 15‐ to 19‐month old group, but there was variation in the trajectories for individual subscales. For example, reduced performance on the VR scale was evident at a much younger age, with the 5‐ to 9‐month old group outperforming all of the older age groups and with evident decline in performance among the older age groups. On the other hand, declines in language performance only became evident among the 15‐ to 19‐month and 20‐ to 24‐month olds. Visual processing and FM skills begin to develop early in infancy (Thompson & Nelson, 2001). On the other hand, while there is evidence of language comprehension from as early as 6‐ to 9‐months (Bergelson & Swingley, 2012), RL skills develop rapidly around 12‐months of age (Swanson et al., 2017). Likewise, children start producing their first words around their first birthday but undergo a vast increase in vocabulary by 24‐ to 36‐months (Swanson et al., 2017). The maturation of early visual and motor skills is fundamental for the development of language ability later on. For example, VR is crucial for label mapping and motor skills are important for providing an infant with opportunities to explore their environment (Iverson, 2010). Thus, the differential onset of decline observed in the different cognitive domains is consistent with the age when milestones in these domains are expected to occur and there appears to be an accumulation in derailment with increasing age.

The regression in cognitive ability becomes starker when the group of infants tested longitudinally is taken into consideration. Firstly, the infants’ cognitive scores significantly decreased within a 3‐month time period. Additionally, this reduction was so pronounced among some participants, that a significant proportion changed from having scores in the average category to falling below average.

The MSEL has been used to map trajectories of cognitive development among a variety of risk groups. Similar declines in cognitive performance are observed within the first 12‐ to 24‐months of life among infants with increased familial risk for ASD (Brian et al., 2014) and infants born pre‐term (Yaari et al., 2018). Taken together, these findings highlight a developmental period where infants at‐risk for cognitive delay begin to exhibit the first behavioural manifestations of adverse outcomes. This is unsurprising, given that this is the period when infants begin to actively engage with their environment and, in turn, environmental demands increase (Karasik, Tamis‐Lemonda, & Adolph, 2014). However, the earlier onset of decline in the VR subscale also suggests that atypicalities actually start to emerge in the perceptual domains first, on which later development cascades.

### 4.2 Association between cognitive performance and measures of growth

The population tested in this study was selected because of the high prevalence of undernutrition and difficulties in growth observed among children in this setting (Hennig et al., 2015; van der Merwe et al., 2013). Within our sample, the average WHZ, HAZ and HCZ scores all fell in the range of normal growth according to WHO criteria (WHO Multicentre Growth Reference Study Group, 2006), however for both HAZ and HCZ from 10‐months onwards the means fell around 1 *z*‐score below the worldwide average. There was evidence of wasting and stunting among a substantial proportion of the children tested. Likewise, all three measures of growth evidenced decline with age from 5‐ to 24‐months.

Consistent with prior research (e.g., Sudfeld et al., 2015), greater HAZ scores were significantly associated with improved MSEL scores, as were WHZ scores. On the contrary, HCZ was not associated with cognitive performance. Stunting (indicated by reduced height for age) is considered to be an indicator of chronic undernutrition, while wasting (reduced weight for age) is more transient and is, thus, an indicator of acute malnutrition (Briend, Khara, & Dolan, 2015). This is perhaps why a majority of studies that examine the association between growth and cognitive development focus primarily on stunting (Perkins et al., 2017; Sudfeld et al., 2015). However, our results contribute to a sparser body of literature (e.g., McDonald et al., 2013), which suggests that reduced weight‐for‐height is also associated with poorer cognitive outcomes. The parallel onset of decline in physical and cognitive development may be partially attributed to the introduction of weaning foods at approximately 4‐ to 6‐months of age (Eriksen et al., 2017; Onofiok & Nnanyelugo, 1998). Lack of nutrient‐rich weaning foods and contamination result in children getting less energy and micronutrient dense foods and increased exposure to pathogens (Arpadi et al., 2009).

Given that HC is posited to be a measure of brain growth (Bartholomeusz, Courchesne, & Karns, 2002) it is surprising that an association between HCZ and MSEL scores was not observed in our sample. However, Lira et al. (2010) report that growth in HC in the first 6‐months of life significantly predicts cognitive outcomes, while subsequent growth does not. Thus, it is possible that the children in our sample were too old to exhibit this association and future research should begin to examine head growth at even younger ages.

There are a multitude of environmental factors that can impact on both infant physical growth and cognitive development (Perkins et al., 2017). In this present study, we did not include measures of other key variables, which limit our ability to make firm conclusions about the nature of this association or any causal inferences. Therefore, we propose that findings from the present study be interpreted as signalling the presence of an association, upon which further research can build. In the aforementioned BRIGHT project, we have included a multitude of measures that are relevant to child physical growth and cognitive development, such as SES, measures of parent–infant interaction and maternal mental health, among others.

### 4.3 Strengths and challenges of the adapted MSEL and implications for future research

Findings from this study suggest that the use of standardized cognitive assessments with infants in LMICs is both feasible and pressing. The adapted MSEL used has several important advantages; it was developed for and tested among children living in a rural setting and was translated into a local language. However, one of the goals of the current study was to highlight the challenges that we encountered while attempting to implement the MSEL into the rural Gambian setting and several key issues emerged.

Infants in this setting are often unfamiliar with the general set‐up used in behavioural testing. For example, toys and books are not readily available in this environment and most play takes place outdoors (Bradley & Corwyn, 2005). Furthermore, children in LMICs are taught to regard adults as authority figures and are discouraged from interacting with strangers (Bradley & Corwyn, 2005). While this has not been studied or documented in The Gambia, in neighbouring rural regions in Senegal, adults are also discouraged from talking to pre‐verbal infants, as cultural beliefs posit that children so young cannot understand speech and that interacting with them is futile (Weber, Fernald, & Diop, 2017). As a consequence of these circumstances, an activity that involves spending a prolonged period indoors, engaging with novel objects on a table top, interacting with an unfamiliar adult experimenter, and being video‐recorded, is an unusual experience for infants in this setting. We observed that the participants were very reticent during testing, even after receiving encouragement from caregivers. The administration time would regularly reach double the expected time from similar aged infants and toddlers undertaking this assessment in our UK studies: this became more apparent as participant age increased. It is possible that girls in this rural environment are even more greatly discouraged from exploring novel environments and interacting with strangers. This could partly explain our finding that girls had reduced performance, which is contradictory to prior literature (Koura et al., 2013). It is also possible that an age‐related decline in GM skills was not observed because physical play outdoors allows children to hone their motor abilities.

Infant non‐compliance and reticence to engage were often reasons why experimenters resorted to parent‐report to score an item. It is encouraging that the number of items scored through parent‐report was not significantly associated with overall scores on the MSEL. Koura et al. (2013) reported that MSEL scores of infants in Benin were significantly associated with a parent‐report measure of cognitive development. Coupled with these findings, our results suggest that parent‐report measures may be useful to supplement behavioural measures in low‐resource settings.

A limitation of this study was that, in the absence of a control group, we relied solely on US‐normed *t*‐scores. The MSEL was originally normed among typically developing children in the US and its validity for testing more diverse populations has been called into question (e.g., Akshoomoff, 2006). Furthermore, since items become more advanced and require more instruction as children get older, the measure could be more culturally sensitive among older children and this could have contributed to the age‐related differences observed in this sample. Conversely, it also is possible that the sensitivity of behavioural measures such as the MSEL increases with age. For example, Nishimura et al. (2016) report that the MSEL was better able to discriminate children with developmental delay when they were older than 12‐months than during the first few months of life. Therefore, we cannot rule out that the decrease in performance among older age groups in our sample could be due to improved effectiveness of the measure with increasing age.

Given the challenges outlined above, it is possible that the standardized scores may not necessarily be suitable criteria against which to compare children from LMICs. While this posed less of a problem in the present study that was examining longitudinal changes in a single population, we propose comparing performance on raw scores when possible. Additionally, if measures such as the MSEL are to be used to identify children at‐risk for cognitive delay, it is crucial to create norms for the specific population, or to identify comparable populations for comparison. While we made every effort to optimize the assessment for this population, it remains difficult to ascertain whether the adaptations of the test materials that we selected were truly optimal for children in this setting. A remaining caveat to the current findings is that it is not possible for us to disentangle whether the decline in cognitive performance with age is also confounded by the appropriateness of this cognitive development scale, for infants and toddlers of this age, within this population.

## CONFLICT OF INTEREST

The authors confirm that they have no conflict of interest.
