# Supplementary material for: Adaptation of the Mullen Scales of Early Learning for use among infants aged 5‐ to 24‐months in rural Gambia
Source: Dev Sci. 2019 Mar 10;22(5):e12808. doi: 10.1111/desc.12808 (PMC6767903; doi:10.1111/desc.12808)
Supplement: Supplementary file 1 [file DESC-22-na-s001.docx]

**Supplementary Material**

In addition to linguistic translations on the MSEL a number of stimuli and test items were found to be inappropriate for the rural Gambian setting and were replaced. Below is a list of adaptations and replacements for items in each subscale.

**Expressive Language:**

Item 15 – Names objects: Since many children are not exposed to picture books, the book was replaced with a toy shoe.

Item 18 - Picture Vocabulary: In this item, the infant is asked to label a series of black and white, drawn images. Multiple objects depicted in these images were either novel to infants in this setting or looked different in their environment. Thus, the following replacements were made:

- Telephone 🡪 Mobile phone
- Bed 🡪 Chair
- Television 🡪 Goat
- House 🡪 Redrawn to resemble houses in West Kiang villages
- Purse 🡪 Donkey
- Clock 🡪 Cup
- Chimney 🡪 Water pump
- Lamp 🡪 Fire
- Paintbrush 🡪 Pencil
- Flower 🡪 Tree

**Receptive Language:**

Item 14 – Identifies objects: The baby doll was replaced by a dark-skinned baby doll.

Item 17 – Follows directions: It was difficult to find a suitable word in Mandinka to translate block. Thus, the block was replaced by a small, plastic donkey, which are very commonplace animals in this area, and most children were familiar with.

Item 22 – Auditory spatial awareness: As with RL item 17, the block was replaced by a toy donkey. For similar reasons, the plastic teddy bear was replaced by a small, plastic toy shoe.

Item 24 – Identifies object function: The scissors used in this item were replaced by a plastic knife.

**Visual Reception:**

Item 11 – Makes object association: The hairbrush was replaced by a small, plastic comb.

Items 17 a and b – Matches object with/without naming: A small, toy donkey was used to replace the toy block.

Item 23 – Memory for one picture: This item involves showing children a picture of a clown, which many children in this setting are unfamiliar with. Therefore, the clown was referred to as the “funny man” or “crazy man”.

**Fine Motor:**

Item 18 – Imitates a 4-block train: The idea that the blocks represented a train was changed to say they represented a car. Additionally, instead of saying “Choo-choo”, experimenters were instructed to say “vroom vroom” so that it sounded like a noise made by a car.
